# A Single Port (SP) Approach Reduces the Risk of Postoperative Complications in Elderly Patients Undergoing Robotic-Assisted Partial Nephrectomy (RAPN)

**DOI:** 10.3390/cancers17081324

**Published:** 2025-04-15

**Authors:** Valerio Santarelli, Fabio Maria Valenzi, Hakan Bahadır Haberal, Luca A. Morgantini, Juan R. Torres-Anguiano, Francesco Del Giudice, Benjamin I. Chung, Alessandro Sciarra, Simone Crivellaro

**Affiliations:** 1 Department of Maternal-Infant and Urological Sciences, “Sapienza” Rome University, Policlinico Umberto I Hospital, 00185 Rome, Italy; 2 Department of Urology, University of Illinois at Chicago, Chicago, IL 60612, USA; 3 Urology Unit, Department of Medico-Surgical Sciences & Biotechnologies, Faculty of Pharmacy & Medicine, Sapienza University of Rome, 04100 Latina, Italy; 4 Department of Urology, Ankara Ataturk Sanatoryum Training and Research Hospital, Ministry of Health, University of Health Sciences, Ankara 06290, Turkey; 5 Department of Urology, Stanford University School of Medicine, Stanford, CA 94305, USA

**Keywords:** Robotic-Assisted Partial Nephrectomy (RAPN), Single Port (SP), ccomplications, elderly

## Abstract

Introduction: In recent years, due to the increasing life expectancy and the growing popularity of nephron sparing strategies (NSS), more elderly and frail patients are undergoing Robotic-Assisted Partial Nephrectomy (RAPN) for the treatment of localized Renal Cell Carcinoma (RCC). A Single Port Approach has demonstrated itself to reduce estimated blood loss (EBL), complication rates, and length of stay (LOS). In the present study, we aim to compare perioperative and postoperative outcomes of SP RAPN and Multi Port (MP) RAPN, with a focus on elderly patients. Materials and Methods: In the study, 293 cN0M0 RCC patients who underwent MP and SP RAPN performed at our institution from 2018 to 2024 were retrospectively reviewed. Clinical, demographic, patient, and tumor characteristics and perioperative variables were assessed across group A (<65 years old) and group B (≥65 years old) patients, as well as MP and SP cohorts. Subsequently, univariate and multivariate regression analyses were carried out, particularly for elderly patients, to evaluate the impact of selected preoperative and perioperative variables on the risk of 30-day postoperative complications. Results: Clinical and demographic characteristics were similar across the MP and SP cohorts of group A and group B. The SP cohort of both group A and group B demonstrated significantly higher rates of extraperitoneal access (75.9% vs. 26.6% for group A, *p* < 0.001 and 81.8% vs. 26.2% for group B, *p* < 0.001), shorter median operative times (186 [142.8–222] min vs. 190 [153–238] min for group A, *p* < 0.001 and 173.5 [143–228] min vs. 206 [178–237] min for group B, *p* < 0.001), and shorter median LOS (0 [0–1] days vs. 2 [1–3] days for group A, *p* < 0.001 and 0 [0,1] days vs. 2 [2–4] days for group B, *p* < 0.001). Moreover, for both groups, an SP approach required significantly longer median ischemia times (21 min [18–31] vs. 20 min [16–24] for group A, *p* = 0.02 and 24.5 min [20–28] vs. 19.5 min [16–26] for group B, *p* = 0.03). However, the SP cohort of group B, despite the lower rate of on-clamp procedures (63.4% vs. 90.5%, *p* = 0.03), demonstrated significantly lower median EBL (50 mL [31–142] vs. 100 mL [50–200], *p* = 0.03). At multivariate analysis, the SP approach was an independent predictor of lower 30-day postoperative complications (OR: 0.2, 95%CI 0.04–0.9, *p* = 0.04). Conclusions: In the present study, elderly patients undergoing SP RAPN did not demonstrate higher complication rates, EBL or hospitalization days compared to their younger counterparts. Moreover, an SP approach was found protective for 30-day postoperative complications. Our results suggest that SP RAPN is feasible and safe in older and frailer patients.

## Simple Summary

In recent years, thanks to the increasing life expectancy and growing adoption of minimally invasive approaches, surgical indications are expanding to older and frailer patients. However, research suggests a higher rate of complications and unfavorable outcomes in the elderly population. The Single Port (SP) surgical system has demonstrated itself to be a safe and feasible alternative to Multi Port (MP) surgical robots. Different studies have already reported lower complication rates and shorter hospital stays for SP Robotic-Assisted Partial Nephrectomy (RAPN) when compared to MP RAPN. In the present study, we reviewed our cohort of 293 SP and MP RAPNs to evaluate perioperative and postoperative outcomes. SP RAPN required shorter operative times and hospital stays. Moreover, we found the SP approach to be a significant and independent predictor of lower 30-day postoperative complications in the elderly population.

## 1. Introduction

Kidney cancer is the 12th most common cancer worldwide, with an age-standardized incidence of 4.6 per 100,000 people [1]. Renal Cell Carcinoma (RCC) makes up most of the kidney cancer. Currently, no large-scale screening method has been accepted and the majority of RCCs are diagnosed incidentally because of unrelated medical interventions [2]. Nonetheless, due to the increasing availability and popularity of abdominal imaging, most kidney cancers are diagnosed in a relatively early and localized stage [3]. For this reason, and thanks to technological surgical advancements, nephron-sparing interventions such as Partial Nephrectomy (PN) are the approach of choice for the majority of kidney lesions requiring treatment [4]. Robotic surgery has become the mainstay for practically every major urological intervention. Current international guidelines recommend surgeons to prefer a nephron-sparing strategy (NSS) whenever technically feasible [5,6]. The advantages of a robotic approach over open or laparoscopic surgery, such as better visualization and enhanced precision, are particularly evident for PNs. Robotic PNs demonstrated reduced blood loss, complication rates, and better oncological results when compared to the laparoscopic approach [7]. The introduction of Single Port (SP) robotic platforms has been the latest surgical innovation in robotic surgery. Despite its relatively recent introduction, the benefits of SP over the standard Multi Port (MP) approach are already becoming evident, particularly for major urological interventions [8,9]. SP Robotic-Assisted Partial Nephrectomy (RAPN), thanks to the single incision site and the possibility of undergoing an extraperitoneal approach, has been demonstrated to reduce intraoperative complications and hospital stays [10,11]. The elderly population, defined as age 65 and older, is a particularly vulnerable group with higher comorbidity and frailty prevalences [12]. With an increasingly aging population, the percentage of people aged 65 or older undergoing surgery is steadily increasing [13]. Particularly in the case of oncological kidney surgery, due to the increasing life expectancy and novel technological advancements, indications of RAPN are expanding to frailer and older patients. Despite the reduced invasiveness and increased precision of robotic-assisted surgery, elderly patients still demonstrate higher intraoperative and postoperative complication rates compared to younger population groups [14,15]. Accordingly, current guidelines propose Active Surveillance (AS) and Tumor Ablation (TA) as viable alternatives for frail or comorbid patients, especially in the case of small renal masses [5,6]. SP robotic-assisted surgery, and in particular SP Robotic-Assisted Radical Prostatectomy (SP-RARP) has been proposed as a safe and feasible procedure for older patients with higher comorbidity scores [16]. Nonetheless, to our knowledge, no previous research has focused on evaluating perioperative and postoperative outcomes of SP RAPN.

The aim of our study is to compare perioperative and postoperative outcomes of SP RAPN and MP RAPN, focusing on elderly patients, ultimately proposing SP RAPN as a feasible and safe procedure for this frail population.

## 2. Materials and Methods

### 2.1. Study Cohort

In this study, 293 patients who underwent SP RAPN and MP RAPN at our institution from 2018 to 2024 were retrospectively reviewed. All data were collected through the electronic database of hospital records (Epic System). Patients’ anthropometric, demographic, and clinical characteristics (i.e., age, gender, race, Body Mass Index (BMI), Charlson Comorbidity Index (CCI), hypertension, diabetes) were collected. All cases were discussed in a Multidisciplinary Team (MDT) session prior to being offered RAPN as the primary therapeutic option. Other relevant therapeutic choices (TA, Radical Nephrectomy, AS), according to the latest American Urological Association (AUA) guidelines at the time of the intervention, were offered and all the included patients chose RAPN and signed the informed consent for the procedure.

Inclusion criteria were the absence of distant metastasis and lymph node involvement at clinical staging, and RAPN performed as a primary therapeutic option with curative intent. Previous External Beam Radiation Therapy (EBRT), chemotherapy, and RAPN performed for other indications rather than curative intent for a solid/cystic lesion suspect for malignancy, were the exclusion criteria. Patients with positive lymph nodes at preoperative clinical staging, and those who underwent Radical Nephrectomy as per preoperative surgical planning were not included. For all the included patients, preoperative clinical staging was performed with either abdominal Multiparametric Resonance Imaging (MRI) or abdominal Computerized Tomography (CT), or both. Tumor side, size, and location were recorded. Renal mass complexity, according to the RENAL nephrometry score, was calculated.

### 2.2. Surgical Procedure

MP RAPNs were performed by two different primary surgeons, while all SP RAPNs were performed by the same primary surgeon. All of the participating surgeons had >10 years of experience with robotic-assisted surgery. All procedures were performed in the setting of a USA-certified Senior Robotic Clinical Fellowship program. MP RAPNs were performed with the patient in a full flank position; SP RAPNs were performed with the patient in either a full flank or supine position. The choice to perform a transperitoneal or retroperitoneal approach was left to the surgeon’s preference and was influenced by patient and tumor characteristics. For patients undergoing MP RAPN, four robotic arms and two assistant ports were adopted. In the case of a transperitoneal approach, pneumoperitoneum was induced using a Verres needle, or alternatively, a mini-open technique. A lower anterior access was adopted in the case of a supine extraperitoneal SP RAPN. When a transperitoneal approach was performed, the colon was medialized in order to access the retroperitoneal space. Subsequently, all procedures were performed using the same standardized technique previously described [17]. The adoption of vascular clamping and ischemia time were recorded. Operative time, intraoperative and 30-day postoperative complications, length of stay (LOS), and estimated blood loss (EBL) were reported. Long-term follow up was not available.

Final histology and pathological stage were reported according to the American Joint Committee on Cancer (AJCC) Guidelines, with all RAPN specimens analyzed by experienced uropathologists at our institution. pT stage, tumor diameter, histological subtype, and surgical margins were reported for the purpose of this study.

### 2.3. Statistical Analysis

Statistical analysis was performed using STATA version 18.1 (Stata Corporation, College Station, TX, USA) and SPSS version 27.0 (IBM SPSS Statistics for Windows, Version 27.0. Armonk, NY, USA: IBM Corp). A two-sided *p* value < 0.05 was considered statistically significant. Descriptive statistics were used to summarize pertinent study information stratified according to age <65 years or ≥65 years and robotic technique (MP or SP). Number of cases, percentages, median, and interquartile (IQR) range were adopted to depict the numerosity of the samples. The Pearson Chi-square test or the Fisher’s exact test, when appropriate, were used to test the association between categorical variables. A Mann–Whitney test or an ANOVA one-way test was adopted to compare quantitative data and pairwise intergroup comparison of variables. Subsequently, a univariate regression model was developed to explore the effect of selected preoperative and intraoperative predictors on the risk of 30-day postoperative complications. Lastly, multivariable logistic regression modeling of clinically relevant variables was adopted to identify independent predictors for 30-day postoperative complications in patients aged ≥65 years.

## 3. Results

### 3.1. Study Cohort Characteristics

Baseline characteristics of the whole population are shown in Table 1. A total of 293 patients were included for final analysis, 207 were 64 years of age or younger (group A) and 86 were 65 years old or older (group B). Median age, BMI, and CCI were, respectively, 58 years (IQR 50–66), 30.5 (IQR 26–35), and 2 (1–3). n =158 (53.9%) patients were male and n = 135 (46.1%) were females. The most represented race was African American (42.7%, n = 125), followed by Hispanic (28.3%, n = 83) and Caucasian (21.5%, n = 63). n = 68 (23.2%); n = 77 (26.3%) patients were current or former smokers and hypertension and diabetes were present in n = 194 (66.2%) and n = 79 (27%) patients, respectively. A left-sided tumor was present in 41.5% (n = 121) of patients and a right-sided lesion occurred in 58.5% (n = 172) of the study cohort. Median tumor diameter and RENAL score were, respectively, 3 cm (2.2–4) and 6 cm (4–7).

In younger patients (group A), anthropometric and demographic characteristics (age, BMI, gender, race) did not significantly vary between the MP and SP cohorts (*p* > 0.05). Comorbidities, including calculated CCI, smoking status, and concomitant hypertension and diabetes rates were similar between MP and SP patients (*p* > 0.05). There were no significant differences in tumor location and diameter. The median RENAL score was significantly higher in the MP group compared to the SP group (6 (IQR 5–8) vs. 5 (IQR 4–6), *p* = 0.01).

Regarding patients aged 65 years or older (group B), the MP and SP cohorts demonstrated similar anthropometric and demographic characteristics (age, BMI, gender, race, *p* > 0.05). Median CCI, as well as smoking status and concomitant hypertension and diabetes rates, were similar between the MP and SP cohort (*p* > 0.05). No significant difference was found in tumor side, diameter, and RENAL score of group B MP and SP cohorts (*p* > 0.05).

For both the MP and SP cohorts, group B demonstrated higher median CCI (*p* = 0.01 and *p* = 0.001, respectively) than group A. In addition, group B patients of the SP cohort had significantly lower median BMI (28.3 (IQR 25–32) vs. 31.5 (IQR 27–38), *p* = 0.01) and higher hypertension rates (81.8% vs. 62%, *p* = 0.02) when compared to group A.

### 3.2. Perioperative and Postoperative Outcomes

Perioperative and postoperative outcomes of the entire population are shown in Table 2.

For both group A and group B, an extraperitoneal access was significantly more frequent in the SP cohorts compared to the MP cohorts (n = 60 (75.9%) extraperitoneal accesses and n = 19 (24.1%) transperitoneal accesses in the SP cohort vs. n = 34 (26.6%) extraperitoneal accesses and n = 94 (73.4%) transperitoneal accesses in the MP cohort for group A, *p* < 0.001 and n = 36 (81.8%) extraperitoneal and n = 8 (18.2%) transperitoneal accesses for the SP cohort vs. n = 11 (26.2%) extraperitoneal and n = 31 (73.8%) transperitoneal accesses for the MP cohort, *p* < 0.001 in group B). Interestingly, for both groups, the SP approach demonstrated significantly lower median operative times (186 (IQR: 142.8–222) vs. 190 (IQR: 153–238), *p* < 0.001 for group A and 173.5 (IQR 143–228) vs. 206 (IQR178–237), *p* < 0.001 for group B). For group B patients, the SP approach demonstrated significantly lower vascular clamping rates (63.4% for the SP cohort and 90.5% for the MP cohort, *p* = 0.03). Nonetheless, when vascular clamping was adopted, the SP approach required significantly longer median ischemia times (21 (IQR18–31) vs. 20 (IQR 16–24) for group A, *p* = 0.02 and 24.5 (IQR 20–28) vs. 19.5 (IQR 16–26), *p* = 0.03 for group B median EBL was significantly lower in the SP cohort of group B patients (50 [31–142] vs. 100 [50–200].

The SP approach demonstrated lower 30-day postoperative complications with superimposable complication rates between the two age groups (n = 23 (18%) for MP vs. n = 6 (7.6%) for SP in group A, and n = 8 (19%) for MP vs. n = 3 (7%) for SP in group B). However, due to the lower sample size, the difference did not reach the significance level in group B (*p* = 0.03 and *p* = 0.07, respectively, for group A and group B). Median LOS was significantly lower in the SP cohorts of both groups (2 [1–3] for MP vs. 0 [0,1] for SP in group A, *p* < 0.001 and 2 [2–4] for MP vs. 0 [0,1] for SP in group B, *p* < 0.001).

Regarding the final pathological analysis, the pT stage and histological subtypes were similar between the SP and MP cohorts of both groups (*p* > 0.05). Interestingly, the SP cohort demonstrated lower positive surgical margin rates (n = 8 (10.1%) for SP vs. n = 30 (23.4%) for MP in group A and n = 4 (9.1%) for SP and n = 8 (19%) for MP in group B), but the difference reached significance only for group A patients (*p* = 0.04).

Perioperative and postoperative outcomes did not significantly differ when comparing the two age groups for both SP and MP patients (Supplementary Table S1). The only exception was median LOS, that was significantly longer in the elderly population of the MP cohort (2 [2–4] vs. 2 [1–3] days, *p* = 0.05).

### 3.3. Risk Assessment for 30-Day Postoperative Complications in the Elderly Group

**Figure 1:**
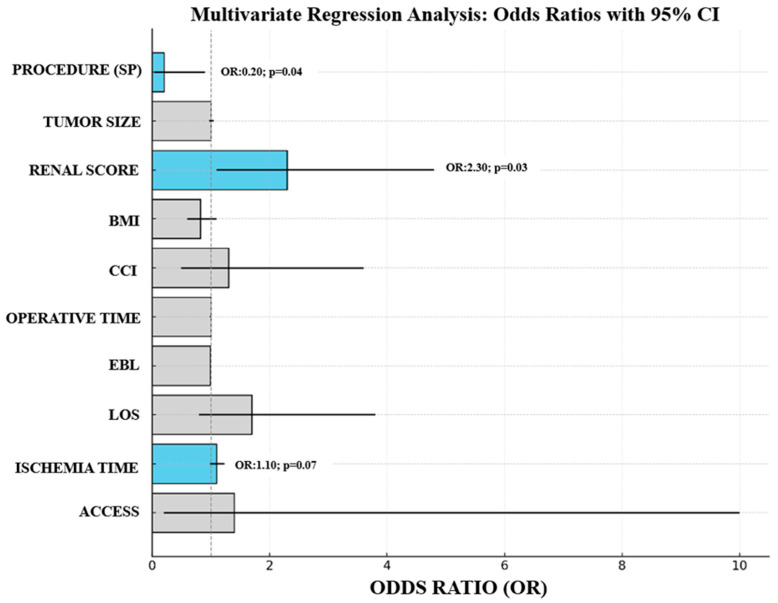
Bar chart representing the Odds Ratios (OR) with 95% Confidence Intervals (CI) for the multivariate regression model exploring the risk of 30-day postoperative complications.

Risk assessment for 30-day postoperative complication rates in group B patients is shown in Table 3. Preoperative anthropometric characteristics such as BMI and CCI did not significantly increase the risk of 30-day postoperative complications. Postoperative complication rate was not influenced by any of the analyzed intraoperative predictors (operation time, ischemia time, EBL, and type of access). The only parameter associated with an increased risk of 30-day postoperative complications was preoperative RENAL score (OR: 1.59, 95%CI 1.03–2.4, *p* = 0.03). As expected, employing the SP surgical robot reduced the risk of 30-day postoperative complications, but the difference did not reach the level of significance at univariate analysis (OR: 0.3, 95%CI 0.08–1.2, *p* = 0.07). At multivariate analysis, the only variable able to independently influence the risk of 30-day postoperative complication rates were the adoption of the SP robotic system (OR: 0.2, 95%CI 0.04–0.9, *p* = 0.04) and the preoperative RENAL score (OR 2.3, 95%CI 1.1–4.8, *p* = 0.03). Of note, the increased risk of complications associated with a prolonged ischemia time was just below the level of significance (OR: 1.1, 95%CI 0.99–1.23, *p* = 0.07). For better visualization, a bar chart depicting the results of multivariate logistic regression is shown in Figure 1.

## 4. Discussion

Current guidelines recommend a nephron-sparing approach over a Radical Nephrectomy (RN) whenever technically feasible [18]. However, while a conservative approach is highly recommended in selected cases, such as a solitary kidney, in the majority of other cases the added advantage of a PN over an RN should always be weighed against the possible added surgical morbidity and complexity [6]. Advanced tumor stage and patient age, high renal mass complexity, multiple comorbidities, renal vein invasion, and reduced homolateral renal function are the main factors limiting the adoption of NSS [19]. PNs demonstrated longer median operative times when compared to RN and prolonged operative times have been associated with higher complication rates, especially in older patients [20,21]. Nonetheless, due to the increasing life expectancy and the technological advancements of modern surgery, a partial approach is becoming the preferred approach, both by surgeons and patients, even for older and frailer populations. The introduction of the SP robotic platform has been a step toward this direction. A single access and robotic arm reduce docking times [8,22]. Moreover, SP RAPN can, in most cases, be performed in a supine position, reducing anesthesia time by nullifying the impact of positioning time and anesthesiologic complications associated with an unnatural position. In addition, the possibility of performing the majority of SP RAPN with an extraperitoneal approach reduces intraoperative complications and challenges associated with the pneumoperitoneum and hostile abdomens, significantly more frequent in older patients who are more likely to have a complex previous surgical history [23,24].

The aim of this study was to assess perioperative and postoperative outcomes of RAPN, performed with both MP and SP approaches, particularly in older patients and to evaluate the plausible advantages of the SP approach in this population. We found that SP RAPN demonstrated significantly lower operative time, both in younger and older patients, contrary to what was previously reported for other major urological interventions. Indeed, when comparing SP and MP Robotic-Assisted Radical Prostatectomy (RARP), previous studies found lower complication rates but at the cost of longer median operative times [25,26,27]. The choice to undergo a transperitoneal or retroperitoneal approach was left to the surgeon’s preference. While tumor and patient characteristics (such as tumor dimension and location, previous abdominal surgery, contraindications to flank position) played a role in determining the approach of choice, the surgeon’s level of confidence with either approach and the surgical system adopted were the major determinants. The MP procedures were performed by two different surgeons and this could have had an impact on the rates of transperitoneal and retroperitoneal procedures. SP RAPNs were performed by a single surgeon. The single incision, 360° rotation, multi-jointed instruments, and fully articulating 3DHD endoscope allow the SP system to efficiently work in narrow spaces such as the retroperitoneum. Accordingly, in our cohort, SP patients were more likely to be operated with a retroperitoneal approach. This strategy, while providing a narrower working space, offers a more direct approach to the kidney and easier identification of relevant anatomical structures, ultimately reducing operative times. When vascular clamping was adopted, the SP RAPN cohort demonstrated significantly longer median ischemia times despite similar mass complexity scores. The difference in median ischemia times, however, was relatively small (1 to 5 min) and did not translate into higher postoperative complication rates, but rather into reduced median estimated blood loss. Nonetheless, longer follow-up is required to fully estimate the possible impact of longer ischemia times on long-term renal function. An SP approach was able to reduce postoperative complication rates in both age categories, but the difference did not reach the level of significance in the older population group, mainly because of the reduced sample size. However, when a multivariable regression model predicting the risk of postoperative complications in older patients was plotted, the SP approach was found to be a significant independent predictor of lower postoperative complications. Notably, when longer ischemia time was considered independently from the robotic approach (MP or SP) in the multivariate regression, it was associated with a nearly significant increased risk of postoperative complications. On the contrary, higher EBL was not found to be a significant predictor of higher complication rates. These results support the advantages of Early Clamp Release (ECR) during PN, reducing ischemia times even at the cost of increased blood loss [28,29,30].

In the present study, we demonstrated that the SP approach is a safe and feasible option to reduce complication rates in elderly patients undergoing RAPN. Our research is not, however, without limitations. Firstly, the present is a retrospective study with a relatively small sample size, particularly for the elderly group. Secondly, not all the operations included in the MP group were performed by the same surgeon of the SP cohort. Despite both surgeons having high expertise in robotic surgery, this variability could have played a role in the presented outcomes. Thirdly, while it is true that all the patients included were offered the possible therapeutic options according to the latest guidelines (mainly AS, PN, RN, and TA) and willingly chose RAPN, our study cohort might not be representative of the entire elderly population. For instance, MDT choices and patient suggestion have probably been influenced by factors such as relevant comorbidities, low performance status, advanced age, and short life expectancy. These highly frail patients might have been more likely to be addressed to TA or AS, even more so when considering the high rate of small T1a masses included in the study, while the included patients were all highly motivated for surgery. Moreover, due to the high rate of small renal masses included in the present studies, results might not apply to larger and more complex masses. Fourthly, due to the relatively low rate of postoperative complications, particularly of serious complications, we were not able to perform multivariate logistic regression models stratified by the Clavien Dindo (CD) score. Nevertheless, while still taking into account the limitations caused by the relatively small sample, we found no significant differences in 30-day postoperative complications’ CD score distribution between the SP and MP cohorts of the elderly group. Lastly, long-term follow-up and renal function evaluation, which could have provided further insight, is lacking from our analysis. Due to the relatively recent introduction of the SP surgical system, data regarding long-term follow-up is mainly lacking in the literature. On one side, we demonstrated a longer ischemia time with the SP approach. Despite some recent studies that appear to suggest otherwise, longer ischemia time has been associated with decreased long-term renal function [31,32]. On the other hand, the supine position and avoiding pneumoperitoneum might help preserve renal function [33]. More research on the long-term impact of an SP retroperitoneal approach on renal function is required.

## 5. Conclusions

As surgical indications are expanding to frailer and older patients, minimally invasive approaches are becoming increasingly popular. In the present study on a total of 293 RAPN patients, we confirmed the advantages of an SP approach in terms of shorter length of stay and reduced complication rates. Moreover, we found that elderly patients undergoing SP RAPN had similar results compared to their younger counterparts, without the need for longer lengths of stays. Finally, particularly for elderly patients, we demonstrated that an SP robotic approach was an independent protective factor for postoperative complications.

## Figures and Tables

**Table 1:** Demographic and clinical characteristic of the study cohort reviewed according to age group and surgical approach.

Variable	TOTAL	GROUP A Age < 65			GROUP B Age ≥ 65			*p* Value Within the MP Group According to Age Group	*p* Value Within the SP Group According to Age Group
		MP	SP	*p* Value	MP	SP	*p* Value		
**N° of cases, n** (**%**)	**293**	128 (61.8%)	79 (38.2%)		42 (48.8%)	44 (51.2%)			
**Age yy, median** (**IQR**)	58 (50–66)	53 (47–59)	55 (47–60)	0.4	71.5 (67–76)	70 (70–73)	0.2	**<0.001**	**<0.001**
**BMI, Kg/m^2^**	30.5 (26–35)	30.1 (26–36)	31.5 (27–38)	0.7	30.3 (26–34)	28.3 (25–32)	0.2	0.4	**0.01**
**Gender, n** (**%**)									
Female	135 (46.1%)	63 (49.2%)	39 (49.4%)	0.9	16 (38.1%)	17 (38.6%)	0.9	0.2	0.3
Male	158 (53.9%)	65 (50.8%)	40 (50.6%)		26 (61.9%)	27 (61.4%)			
**CCI n, median** (**IQR**)	2 (1–3)	2 (1–2)	2 (1–3)	0.2	4 (3–5)	4 (3–6)	0.1	**0.01**	**0.001**
**Race, n** (**%**)									
African American	125 (42.7%)	51 (39.8%)	34 (43%)	0.5	19 (45.2%)	21 (47.7%)	0.7	0.2	0.1
Caucasian	63 (21.5%)	28 (21.9%)	16 (20.3%)		8 (19%)	11 (25%)			
Hispanic	83 (28.3%)	40 (31.3%)	19 (24.1%)		13 (31%)	11 (25%)			
Asian	8 (2.7%)	2 (1.6%)	3 (3.8%)		2 (4.8%)	1 (2.3%)			
Other	14 (4.8%)	7 (5.5%)	7 (8.9%)		0	0			
**Smoking, n** (**%**)									
No	148 (50.5%)	69 (53.9%)	37 (46.8%)	0.25	25 (59.2%)	17 (38.6%)	0.09	0.06	0.3
Current	68 (23.2%)	33 (25.8%)	21 (26.6%)		4 (9.5%)	10 (22.7%)			
Former	77 (26.3%)	26 (20.3%)	21 (26.6%)		13 (31%)	17 (38.6%)			
**Hypertension, n** (**%**)									
No	99 (33.8%)	49 (38.3%)	30 (38%)	0.9	12 (28.6%)	8 (18.2%)	0.2	0.2	**0.02**
Yes	194 (66.2%)	79 (61.7%)	49 (62%)		30 (71.4%)	36 (81.8%)			
**Diabetes, n** (**%**)									
No	214 (73%)	99 (77.3%)	57 (72.2%)	0.4	30 (71.4%)	28 (63.6%)	0.4	0.4	0.3
Yes	79 (27%)	29 (22.7%)	22 (27.8%)		12 (28.6%)	16 (36.4%)			
**Renal score, median** (**IQR**)	6 (4–7)	6 (5–8)	5 (4–6)	**0.01**	6 (5–8)	5 (4–7)	0.1	0.6	0.4
**Tumor diameter cm, median** (**IQR**)	3 (2.2–4)	2.9 (2–4)	3 (2.3–4.1)	0.3	2.8 (2–3.6)	3 (2.4–4.1)	0.3	0.9	0.8
**Side, n** (**%**)									
Right	172 (58.5%)	67 (52.3%)	51 (64.1%)	0.1	25 (59.5%)	29 (65.9%)	0.6	0.4	0.8
Left	121 (41.5%)	61 (47.7%)	28 (35.9%)		17 (40.5%)	15 (34.1%)			

MP: Multi Port; SP: Single Port; IQR: Interquartile Range; BMI: Body Mass Index; CCI: Charlson Comorbidity Index.

**Table 2:** Perioperative, postoperative, and pathological characteristic of the study cohort reviewed according to age group and surgical approach.

Variable	TOTAL	GROUP A Age < 65			GROUP B Age ≥ 65		
		MP	SP		MP	SP	
**N° of cases, n** (**%**)	**293**	128	79		42	44	
**Access, n** (**%**)							
Transperitoneal	152 (51.9%)	94 (73.4%)	19 (24.1%)	**<0.001**	31 (73.8%)	8 (18.2%)	**<0.001**
Extraperitoneal	141 (48.1%)	34 (26.6%)	60 (75.9%)		11 (26.2%)	36 (81.8%)	
**Operative time min, median** (**IQR**)	189.5 (152–232)	190 (153–238)	186 (142.8–222)	**<0.001**	206 (178–237)	173.5 (143–228)	**<0.001**
**Clamping, n** (**%**)							
Off-clamp	61 (20.8%)	22 (17.2%)	19 (24.1%)	0.2	4 (9.5%)	16 (36.4%)	**0.03**
On-clamp	232 (79.2%)	106 (82.8%)	60 (75.9%)		38 (90.5%)	28 (63.4%)	
**Ischemia time, median** (**IQR**)	20 (17–26)	20 (16–24)	21 (18–31)	**0.02**	19.5 (16–26)	24.5 (20–28)	**0.03**
**EBL ml, median** (**IQR**)	100 (50–200)	100 (50–200)	50 (47–200)	0.3	100 (50–200)	50 (31–142)	**0.04**
**Intraoperative complications, n** (**%**)							
No	280 (95.6%)	123 (96.1%)	77 (97.5%)	0.5	40 (95.2%)	40 (90.9%)	0.4
Yes	13 (4.4%)	5 (3.9%)	2 (2.5%)		2 (4.8%)	4 (9.1%)	
**LOS days, median** (**IQR**)	2 (1–2)	2 (1–3)	0 (0–1)	**<0.001**	2 (2–4)	0 (0–1)	**<0.001**
**30-day postoperative complications, n** (**%**)							
No	253 (86.3%)	105 (82%)	73 (92.4%)	**0.03**	34 (81%)	41 (93%)	0.07
Yes	40 (13.7%)	23 (18%)	6 (7.6%)		8 (19%)	3 (7%)	
**30-day postoperative complications CD, n** (**%**)							
1	18 (45%)	10 (43.5%)	3 (50%)	**0.05**	4 (50%)	1 (33.3%)	0.3
2	11 (27.5%)	10 (43.5%)	0		1 (12.5%)	0	
3	9 (22.5%)	2 (8.7%)	3 (50%)		3 (37.5%)	1 (33.3%)	
4	2 (5%)	1 (4.3%)	0		0	1 (33.3%)	
5							
**Histology**							
Benign	56 (19.2%)	22 (17.2%)	16 (20.5%)	0.9	12 (28.6%)	6 (14%)	0.4
Clear cell RCC	150 (51.5%)	66 (51.6%)	41 (52.6%)		19 (45.2%)	24 (55.8%)	
Papillary RCC	56 (19.2%)	24 (18.8%)	13 (16.7%)		9 (21.4%)	10 (23.3%)	
Other	29 (9.8%)	16 (12.5%)	8 (10%)		2 (4.8%)	3 (7%)	
**pT stage, n** (**%**)							
T1a	172 (75.4%)	75 (74.3%)	45 (76.3%)	0.4	25 (80.6%)	27 (73%)	0.15
T1b	39 (17.1%)	17 (16.8%)	11 (18.6%)		5 (16.1%)	6 (16.2%)	
T2a	4 (1.8%)	2 (2%)	0		1 (3.2%)	1 (2.7%)	
T2b	0	0	0		0	0	
T3a	12 (4.1%)	7 (6.9%)	2 (3.4%)		0	3 (8.1%)	
T3b	0	0	0		0	0	
T4	1 (0.4%)	0	1 (1.7%)		0	0	
**Positive surgical margins, n** (**%**)							
No	243 (82.6%)	98 (76.6%)	71 (89.9%)	**0.04**	34 (81%)	40 (90.9%)	0.2
Yes	50 (17.3%)	30 (23.4%)	8 (10.1%)		8 (19%)	4 (9.1%)	

MP: Multi Port; SP: Single Port; IQR: Interquartile Range; EBL: estimated blood loss; LOS: length of stay; CD: Clavien Dindo; RCC: Renal Cell Carcinoma.

**Table 3:** Univariate and multivariate regression models exploring the risk of 30-day postoperative complications on the basis of multiple preoperative predictors.

	UNIVARIATE			MULTIVARIATE		
Variable	OR	95%CI	*p* Value	OR	95%CI	*p* Value
Procedure (SP)	0.3	0.08–1.2	0.07	0.2	0.04–0.9	**0.04**
Tumor size	1.01	0.97–1.1	0.6	1.0	0.97–1.05	0.7
RENAL score	1.59	1.03–2.4	**0.03**	2.3	1.1–4.8	**0.03**
BMI	0.9	0.8–1.02	0.1	0.82	0.6–1.1	0.2
CCI	1.05	0.7–1.4	0.8	1.3	0.5–3.6	0.5
Operation time	1.0	0.99–1.01	0.9	1.0	0.98–1.01	0.3
EBL	1.0	0.99–1.01	0.8	0.99	0.98–1.01	0.7
Ischemia time	1.03	0.99–1.1	0.08	1.1	0.99–1.23	**0.07**
Access (retroperitoneal)	1.5	0.4–5	0.6	1.4	0.2–10	0.7

OR: Odds Ratio; 95%CI: 95% Confidence Interval; SP: Single Port; BMI: Body Mass Index; CCI: Charlson Comorbidity Index; EBL: estimated blood loss; LOS: length of stay.

## Data Availability

The data presented in this study are available on request from the corresponding author.

## Supplementary Materials

The following supporting information can be downloaded at: https://www.mdpi.com/article/10.3390/cancers17081324/s1, Table S1: Perioperative, postoperative and pathological characteristic of the study cohort reviewed according to surgical approach and age group.
