# Supplementary material for: A Single Port (SP) Approach Reduces the Risk of Postoperative Complications in Elderly Patients Undergoing Robotic-Assisted Partial Nephrectomy (RAPN)
Source: Cancers (Basel). 2025 Apr 15;17(8):1324. doi: 10.3390/cancers17081324 (PMC12026311; doi:10.3390/cancers17081324)
Supplement: Supplementary file 1 [file cancers-17-01324-s001.zip › cancers-3567275-supplementary.pdf]

Supplementary table 1. Perioperative, postoperative and pathological characteristic of the study cohort reviewed according to surgical approach and age group.

Variable	TOTAL	MP			SP		
		Group A	Group B	P value	Group A	Group B	P value
**N° of cases, n (%)**	**293**	128	42		79	44	
**Access, n (%)**							
Transperitoneal	152 (51.9%)	94 (73.4%)	31 (73.8%)	0.9	19 (24.1%)	8 (18.2%)	0.5
Extraperitoneal	141 (48.1%)	34 (26.6%)	11 (26.2%)		60 (75.9%)	36 (81.8%)	
**Operative Time min, median (IQR)**	189.5 (152-232)	190 (153-238)	206 (178-237)	0.2	186 (142.8-222)	173.5 (143-228)	0.9
**Clamping, n (%)**							
Off-clamp	61 (20.8%)	22 (17.2%)	4 (9.5%)	0.2	19 (24.1%)	16 (36.4%)	0.2
On-Clamp	232 (79.2%)	106 (82.8%)	38 (90.5%)		60 (75.9%)	28 (63.4%)	
**Ischemia time, median (IQR)**	20 (17-26)	20 (16-24)	19.5 (16-26)	0.9	21 (18-31)	24.5 (20-28)	0.2
**EBL ml, median (IQR)**	100 (50-200)	100 (50-200)	100 (50-200)	0.2	50 (47-200)	50 (31-142)	0.8
**Intraoperative Complications, n (%)**							
No	280 (95.6%)	123 (96.1%)	40 (95.2%)	0.8	77 (97.5%)	40 (90.9%)	0.15
Yes	13 (4.4%)	5 (3.9%)	2 (4.8%)		2 (2.5%)	4 (9.1%)	
**LOS days, median (IQR)**	2 (1-2)	2 (1-3)	2 (2-4)	**0.05**	0 (0-1)	0 (0-1)	0.5
**30-day postoperative complications, n (%)**							
No	253 (86.3%)	105 (82%)	34 (81%)	0.8	73 (92.4%)	41 (93%)	0.9
Yes	40 (13.7%)	23 (18%)	8 (19%)		6 (7.6%)	3 (7%)	
**30-day postoperative complications CD, n (%)**							
1	18 (45%)	10 (43.5%)	4 (50%)	0.15	3 (50%)	1 (33.3%)	0.3
2	11 (27.5%)	10 (43.5%)	1 (12.5%)		0	0	
3	9 (22.5%)	2 (8.7%)	3 (37.5%)		3 (50%)	1 (33.3%)	
4	2 (5%)	1 (4.3%)	0		0	1 (33.3%)	
5							
**Histology**							
Benign	56 (19.2%)	22 (17.2%)	12 (28.6%)	0.3	16 (20.5%)	6 (14%)	0.6
Clear Cell RCC	150 (51.5%)	66 (51.6%)	19 (45.2%)		41 (52.6%)	24 (55.8%)	

Papillary RCC	56 (19.2%)	24 (18.8%)	9 (21.4%)		13 (16.7%)	10 (23.3%)	
Other	29 (9.8%)	16 (12.5%)	2 (4.8%)		8 (10%)	3 (7%)	
**pT stage, n (%)**							
T1a	172 (75.4%)	75 (74.3%)	25 (80.6%)	0.4	45 (76.3%)	27 (73%)	0.5
T1b	39 (17.1%)	17 (16.8%)	5 (16.1%)		11 (18.6%)	6 (16.2%)	
T2a	4 (1.8%)	2 (2%)	1 (3.2%)		0	1 (2.7%)	
T2b	0	0	0		0	0	
T3a	12 (4.1%)	7 (6.9%)	0		2 (3.4%)	3 (8.1%)	
T3b	0	0	0		0	0	
T4	1 (0.4%)	0	0		1 (1.7%)	0	
**Positive Surgical margins, n (%)**							
No	243 (82.6%)	98 (76.6%)	34 (81%)	0.7	71 (89.9%)	40 (90.9%)	0.8
Yes	50 (17.3%)	30 (23.4%)	8 (19%)		8 (10.1%)	4 (9.1%)	

MP: Multi Port; **SP**: Single Port; **IQR** : Interquartile Range ; **EBL** : Estimated Blood Loss ; **LOS** : Length of Stay ; **CD** : Clavien Dindo; **RCC**: Renal Cell Carcinoma.
